# GBD 2015: migraine is the third cause of disability in under 50s

**DOI:** 10.1186/s10194-016-0699-5

**Published:** 2016-11-14

**Authors:** Timothy J. Steiner, Lars J. Stovner, Theo Vos

**Affiliations:** 1 Department of Neuroscience, Norwegian University of Science and Technology (NTNU), Edvard Griegs Gate, Trondheim, Norway; 2 Division of Brain Sciences, Imperial College London, London, UK; 3 Norwegian Advisory Unit on Headache, Department of Neurology and Clinical Neurophysiology, St Olavs University Hospital, Trondheim, Norway; 4 Institute of Health Metrics and Evaluation (IHME), University of Washington, Seattle, WA USA

**Keywords:** Headache disorders, Migraine, Tension-type headache, Medication-overuse headache, Burden of disease, Disability, Public health, Global Burden of Disease study, Global Campaign against Headache

## How much headache is there in the world?

The answer depends on how headache is measured. The question, however, is an important one. It is at the basis of health policy, prioritisation and the due allocation of health resources to headache care and the mitigation of its clinical sequelae. From this perspective, prevalence alone is not highly informative: it is the burdens arising from headache disorders that dictate their impact on public health. These burdens are multiple, diverse and partly invisible [1]. Methods do not yet exist to measure them all [1], but the focus meanwhile has been on disability. It is to this, primarily, that health, quality of life, productivity and financial security are hostage.

So how much headache-related disability is there in the world? Estimates of disability due to disease are a principal objective of the Global Burden of Disease (GBD) studies, performed reiteratively since 1990 and described now as “the most comprehensive worldwide observational epidemiological study to date” [2]. GBD 1990 was initiated by the World Bank and GBD 2000 by the World Health Organization; subsequently, GBD has been led by the Institute for Health Metrics and Evaluation [3], and financially supported by the Bill and Melinda Gates Foundation. GBD uses a number of metrics: among these, disability is measured in years lived with disability (YLDs) and early mortality in years of life lost (YLLs); disability-adjusted life years (DALYs) are the summation of YLDs and YLLs. Migraine first featured in GBD 2000 [4], and over 13 years ascended the ranks of top causes of YLDs worldwide, from 19^th^ in GBD 2000 [4] to seventh in GBD 2010 [5, 6] and sixth in GBD 2013 [7, 8]. Meanwhile, of the other headache disorders of public-health importance, tension-type headache (TTH) was introduced in GBD 2010 [5] and medication-overuse headache (MOH) in GBD 2013 (and ranked 18^th^ highest cause of YLDs) [7]. GBD 2013 established headache disorders collectively as the third highest cause of YLDs [8].

The rise of migraine over these years is not indicative of increasing prevalence. While GBD is dependent on data from the entire world, headache epidemiology is a still-developing science [9]. Very large knowledge gaps existed in 2000, particularly in regions outside the Americas and Western Europe [10]. Filling these gaps became the first priority of the Global Campaign against Headache after its launch in 2004 [11–13]. Collaborating in all subsequent GBD studies, the Global Campaign has informed them by conducting new population-based surveys in Georgia, Russia, China, Nepal, South India, Saudi Arabia, Pakistan, Zambia, Ethiopia and Morocco [14]. This concerted data-collection effort has allowed much better estimates from GBD 2010 onwards. With empirical data replacing many of the assumptions underlying the earlier GBD estimates, and an approach to YLD calculation based on prevalence rather than incidence and duration as in GBD 2000, estimates became possible by country rather than by large world regions.

GBD 2015 has now been published [15, 16]. In this latest iteration, a more systematic hierarchy has been adopted in the grouping of related causes of DALYs. Non-communicable disorders, at level 1, include neurological disorders at level 2; within the latter reside the individual headache disorders (migraine, TTH and MOH) at both levels 3 and 4. Future iterations of GBD may more logically group the headache disorders together at level 3, as we have done in the following analysis.

Headache disorders account for more DALYs than all other neurological disorders combined (including dementias), despite having no association with mortality [16]. Because of this latter fact, the comparative impact of headache disorders on public health is better indicated by YLDs, taking disability but not mortality into account. At level 3, headache disorders occupy sixth place among the leading causes of disability, varying between 3^rd^ and 7^th^ in regions around the world [15] (Table 1). While this demotion by three places compared with GBD 2013 [8] may appear as a diminution, this is not so: successive iterations of GBD, increasingly better informed, have shown total YLDs attributed to headache disorders rising consistently. Rather, at level 3, headache disorders are displaced by GBD 2015 groupings of low back and neck pain, which clearly takes top place, sense organ diseases in second place and skin diseases in fifth [15].

**Table 1:** Years lived with disability (YLDs) attributed to all headache disorders^a^ by gender, age and world region

Region	Gender	Age range (years)	YLDs per 100,000	% of total	Rank^b^
Global	Both	All	601	5.61	6
		15–49	813	8.13	3
		50–69	732	4.67	6
	M	All	438	4.35	7
		15–49	597	6.49	3
		50–69	516	3.25	6
	F	All	767	6.76	5
		15–49	1,036	9.59	3
		50–69	940	5.58	6
Western Europe	Both	All	783	6.32	4
Central and Eastern Europe and Central Asia			778	6.41	4
North Africa and Middle East			702	6.80	3
South Asia			730	6.55	5
SE and East Asia and Oceania			443	4.48	7
High-income Asia Pacific			607	5.34	5
High-income North America			646	5.01	6
Latin America and Caribbean			688	6.76	4
Sub-Saharan Africa			449	4.27	6

^a^Migraine, tension-type headache and medication-overuse headache

^b^among the top level-3 causes of disability in GBD 2015

At level 4, migraine on its own is the 21^st^ leading cause of DALYs worldwide, tenth in Western Europe and sixth worldwide in the age group 25–39 years [16]. Migraine, TTH and MOH are all diseases mainly affecting young adults: in terms of YLDs, they remain collectively in third place among level-3 causes in both males and females aged 15–49 years [15] (Table 1). This is due largely to migraine, which on its own, at level 4, is third in this age group in both genders (Table 2). In present estimates, migraine, estimated to affect 959 million people worldwide, emerges by a long distance as the most disabling headache disorder at population level [15]. TTH, despite being the second most prevalent disorder in the world (behind dental caries) [7], adds much less to population disability estimates because a low disability weight of 0.036 (on a scale of 0–1) is accorded to the headache of TTH compared with 0.434 for the ictal state of migraine [17]. MOH is recognised as far more disabling at an individual level but, with its prevalence still very poorly estimated in many regions [18, 19], it stays ranked as 18^th^ cause of YLDs in GBD 2015 [15].

**Table 2:** Years lived with disability (YLDs) attributed to migraine by gender, age, world region and country income

Region	Gender	Age range (years)	YLDs per 100,000	% of total	Rank^a^
Global	Both	All	446	4.17	7
		15–49	615	6.16	3
		50–69	495	3.03	8
	M	All	311	3.09	8
		15–49	432	4.70	3
		50–69	324	2.05	13
	F	All	584	5.15	4
		15–49	805	7.46	3
		50–69	659	3.91	7
Western Europe	Both	All	580	4.69	5
Central and Eastern Europe and Central Asia			515	4.25	6
North Africa and Middle East			498	4.83	5
South Asia			569	5.11	5
SE and East Asia and Oceania			334	3.39	8
High-income Asia Pacific			434	3.82	5
High-income North America			473	3.68	8
Latin America and Caribbean			497	4.89	7
Sub-Saharan Africa			312	3.12	6
All low-income countries	Both	All	314	3.18	6
	M		227	2.41	8
	F		400	3.88	5
All high-income countries	Both	All	502	4.09	6
	M		296	2.62	7
	F		700	5.31	4

^a^among the top level-4 causes of disability in GBD 2015

There can be no doubt that migraine is a major contributor to public ill health in all countries, climes and cultures. Table 2 shows it consistently ranked fifth to eighth among the top causes of disability in all world regions. Further, the notion that migraine is a disease preferentially affecting rich industrialised nations is dispelled by the comparison in Table 2 between low- and high-income countries. It is worth adding here that GBD currently considers only the disability burden associated with the ictal state of headache disorders, whereas there is evidence of interictal burden in a considerable proportion of people with migraine and in a small proportion of people with TTH [20]. The significance of interictal burden is that, although it may be at relatively low level, it is present for longer periods of time than ictal burden. With new data indicating that interictal disability in headache disorders is real and measurable [20], future iterations of GBD should consider adding this component of disability.

Where to next? If future GBD studies group the headache disorders together at level 3, they might consider including neck pain in this grouping rather than with low back pain: the last is markedly different from neck pain not only in its clinical sequelae but also aetiologically, whereas how much neck pain is actually secondary to headache? Meanwhile it is still not known how much headache – or headache-related disability – there is in the world. There are still large data gaps to be filled, particularly with regard to MOH, which only recently has been included as a separate entity in population-based studies [18, 19]. The Global Campaign has further studies underway or planned: in Guatemala, Peru, Nigeria, Uganda, Kuwait, North India, Sri Lanka and Mongolia [14]. GBD estimates of global averages properly take into account all available data, but published surveys are up to 30 years old, and use a variety of ascertainment methods [10]. Crude adjustments are relied upon to correct for measurement biases arising from less than ideal study methods or questionable case definitions. Global Campaign studies use standardised and higher quality methodology [9]. With the single exception of China, all of them so far performed have produced national estimates greater than GBD’s mean global estimates [6, 8]. As the new studies continue to feed data into future iterations of GBD, it is inevitable that the proportion of global disability correctly attributed to headache will continue to rise.

The importance of this measure is that it serves as a needs assessment, marking the existence of a very large burden of ill health to inform health policy in countries and regions the world over. GBD measures disease burden as it is – alleviated by whatever treatments are made available. The persistence of this heavy headache burden is a clear signal of continuing health-care failures that must be addressed [21, 22].
